# An Investigation on the Application of Pulsed Electrodialysis Reversal in Whey Desalination

**DOI:** 10.3390/ijms20081918

**Published:** 2019-04-18

**Authors:** Arthur Merkel, Amir M. Ashrafi

**Affiliations:** MemBrain s. r. o. (Membrane Innovation Center), Pod Vinicí 87, 471 27 Stráž pod Ralskem, Czech Republic; Arthur.merkel@membrain.cz

**Keywords:** whey, electrodialysis, pulsed electric field, pulsed electrodialysis reversal, fouling

## Abstract

Electrodialysis (ED) is frequently used in the desalination of whey. However, the fouling onto the membrane surface decreases the electrodialysis efficiency. Pulsed Electrodialysis Reversal (PER), in which short pulses of reverse polarity are applied, is expected to decrease the fouling onto membrane surface during ED. Three (PER) regimes were applied in the desalination of acid whey (pH ≤ 5) to study their effects on the membrane fouling and the ED efficiency. The PER regimes were compared to the conventional ED as the control. For each regime, two consecutive runs were performed without any cleaning step in-between to intensify the fouling. After the second run, the membranes were subjected to the Scanning electron microscope (SEM) imaging and contact angle measurement to investigate the fouling on the membrane surface in different regimes. The ED parameters in the case of conventional ED were almost the same in the first and the second runs. However, the parameters related to the ED efficiency including ED capacity, ash transfer, and ED time, were deteriorated when the PER regimes were applied. The contact angle values indicated that the fouling on the diluate side of anion exchange membranes was more intensified in conventional ED compared to the PER regimes. The SEM images also showed that the fouling on the diluate side of both cation and anion exchange membranes under PER regimes was reduced in respect to the conventional ED. However, the back transfer to the diluate compartment when the reverse pulse was applied is dominant and lowers the ED efficiency slightly when the PER is applied.

## 1. Introduction

Whey is a by-product of cheese and curd production. It is separated from casein during the manufacture of cheese or casein. Due to its high content of proteins, minerals, vitamins, and lactose, it is a potential source of nutrients. However, in its normal form, whey is not considered as foodstuff due to its high salt content. Whey is categorized into sweet whey (pH is around 6), that is produced from rennet-coagulated casein or cheese, and acid whey (pH ≤ 5) that is produced from mineral or lactic acid-coagulated casein. Considering its content of proteins and vitamins in the natural functional form, whey is a valuable product which can be used as an additive in baby food, cheese products, and candies [1,2,3,4]. Therefore, a method to desalinate the whey and utilize the demineralized whey is in high demand. It is worth noting that the decomposition of the proteins and vitamins must be avoided during its demineralization process. Membrane processes including pressure-driven and electrically-driven membranes are two main solutions for the desalination of whey. Considering that the ED is based on the electrical voltage difference as the driving force, it is a more efficient method for demineralization, particularly in the case of charged ionic species with a small size [5]. However, the application of ED is accompanied by inherent limitations, including concentration polarization and fouling on the membranes [6].

Fouling on membranes is a serious problem in which ion exchange membranes are fouled by ionic materials of medium molecular weight such as ionic surface active agents having the charge opposite to the fixed charges of the membrane. Scaling is another type of fouling that occurs when salts of limited solubility precipitate from the concentrate stream as scale [6]. It must be mentioned that pH change caused by the water splitting in solution—membrane interface results in scaling of the ions with low solubility on the membrane surface [7].

The pore size of the ion exchange membrane is approximated to be 10A; therefore, ions of medium molecular weight permeate with difficulty through the membrane. Consequently, the electrical resistance of the membrane increases during electrodialysis due to clogging of the membrane pores with the medium molecular weight ions [8]. To remove the fouling from the membrane, the cleaning process or even the membrane replacement is required which may cost about 40%–50% in electro-membrane processes [6].

The conventional method to partially avoid the fouling during ED is the reversal of the concentrate and diluate streams. The modification of the membranes used in ED is another strategy to avoid fouling [9,10,11]. Furthermore, using the cleaning agents can also be applied to remove the film attached to the membrane surface during ED [12]. Due to the complexity of the described methods, it is desired to find an alternative method which is easy to perform.

The use of pulsed electric field (PEF) was shown to be an alternative for fouling prevention. The PEF procedure consists of application of consecutive pulse and pause lapses of a certain duration (Ton/Toff).

The use of PEF, particularly when the pause period is extended results in the electrophoretic movement of the substances that form the screening film on the membrane surface. Furthermore, the water splitting in the solution—membrane interface caused by concentration polarization reduces due to restoration during the pause period. Consequently, the scaling of ions with low solubility also decreases. However, Sistat et al. explained that the efficiency in PEF relies on the frequency of applied potential where the efficiency in PEF increases with increasing of the frequency of the potential [13,14]. Desalination of whey has been of great importance in the food industry and therefore many studies have been carried out in this field [15,16,17,18]. The effect of the PEF on the electrodialysis of acid whey to remove the lactate was also investigated [16]. Dufton et al. applied the PEF for the desalination of acid whey and confirmed its antifouling effect. However, the time of the desalination was increased in PEF by several times to reduce the fouling on to the membrane surface [15]. In pulsed electrodialysis reversal (PER) short pulses of reverse polarity are applied instead of a long pause period in PEF. Thus, it is expected that the short period of reverse pulses the ions and the film on the membrane re-dissolves in the solution. In addition, because of the short period of the reverse pulse the ED process will not be much longer compared to the conventional ED. The change of polarity occurs without reversal of diluate and concentrate streams (in contrary to electrodialysis reversal) [6,13,19,20,21,22,23]. This work aimed to study the effect of PER on the membrane fouling in electrodialysis of acid whey.

## 2. Results and Discussion

### 2.1. Electrodialysis

**Figure 1:**
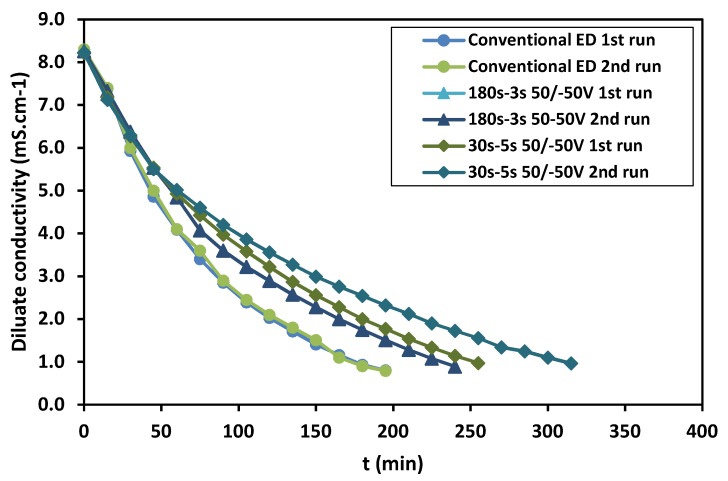
The change in diluate conductivity in different regimes.

Three potential regimes were applied in ED and compared in terms of the efficiency (the regimes are defined as, regime *I*: conventional ED; 50 V applied on the membrane stack, regime *II*: 50 V applied for 180 s and then −50 V for 3 s, regime *III*: 50 V applied for 30 s and then −50 V for 5 s). The change in the diluate conductivity during ED with different regimes is shown in Figure 1. As seen, in conventional ED the time required to reach the desired conductivity in diluate is the same in the first and the second runs. In contrast, in the case of PER more time is required to achieve a given conductivity in the second run compared to that of the first runs. The ED parameters for different regimes are also represented in Table 1. As can be observed, the ED parameters in the case of conventional ED are almost the same in the first and the second runs. However, the parameters related to the ED efficiency including ED capacity, ash transfer, and ED time, deteriorated when the PER regimes were applied. Since the ED continued to obtain a certain conductivity in diluate, the degree of the demineralization value is almost the same in all the applied regimes. The electrodialysis capacity of PER regimes is reduced compared to that of conventional ED, indicating that more time is required to achieve a given degree of the demineralization in PER regimes. The obtained values of the ash transfer rate and the energy consumption also show that to achieve a given diluate conductivity, in PER more energy must be consumed due to lower ash transfer. Recalling only the potential regime was different in the ED processes, as the deteriorated efficiency of the PER regimes could be due to either the fouling on the membrane surface or the back transfer to the dilute when the reverse pulse was applied. The highest difference between the first and the second run was observed in the case of regime III in which the ratio of T_working_/T_reverse_ was the least and the reverse pulse duration was the highest. Evidently, by increasing the duration of the reverse pulse, the back transfer of ions to the diluate increases. The change in current on the membrane stack and the pH change in diluate and concentrate during electrodialysis are provided in Supplementary Materials (Figures S1 and S2 respectively).

### 2.2. Fouling Analysis

**Figure 2:**
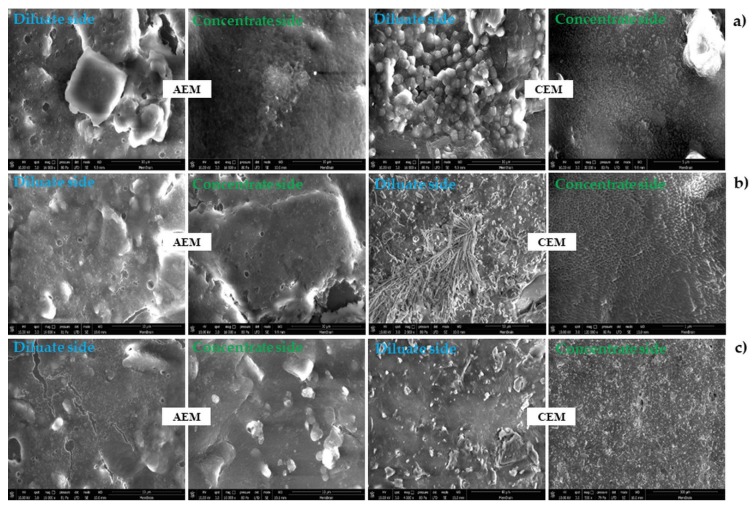
SEM images of the membranes after different regimes, (**a**) regime *I,* (**b**) regime *II*, (**c**) regime *III*, (AEM = anion exchange membrane, CEM = cation exchange membrane). 16000 × magnification

As shown in the SEM images (Figure 2), obviously when the pulsed regimes were applied the fouling decreased on the membrane surface. In particular, the film attached to the diluate sides of both AEM and CEM can be observed. The observed film contains particles which can be the organic molecules as well as the scaling layer. In our previous work we analyzed film attached to the membrane after the whey demineralization and it was found out that the film contains mainly Ca^2+^ and Mg^2+^ and Al^3+^ (the ions with less solubility natural pH). With the electrodialysis process proceeding, the ion concentration near the diluate side of the membrane becomes zero, causing the water splitting and generation of OH^−^ and H_3_O^+^ ions. Consequently, the pH changes which brings about the scaling of minerals (multivalent ions) and fouling of organic molecules on the membranes including amino acids, vitamins, and polypeptides existing in the whey. The organic fouling also might be caused because of sorption of whey components including the residue of whey protein after nanofiltration, amino acids, and polypeptides [19]. In our previous work the scaling on the ion exchange membranes was analyzed and it was found that the scaling is mainly composed of sulfate and phosphate of Ca^2+^ and Mg^2+^ cations [24]. Thus, it is expected that during the reversal of the applied potential, the precipitated ions on the membrane surface partially detach from the membrane surface and dissolve in the feed.

**Figure 3:**
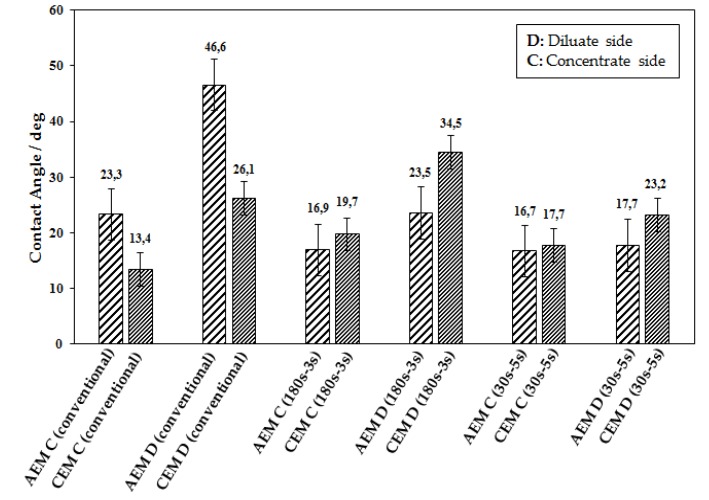
The values of the measured contact angles on the membranes surface after each ED of whey with different regimes.

Therefore, the restoration of the ion concentration at the membrane interfaces is expected to occur in PER electrodialysis during the reverse pulse, resulting in a decrease in scaling and fouling. The values of the membranes contact angle after the ED are represented in Figure 3. The contact angles measurement allows measuring of the surface hydrophobicity. The surface hydrophobicity of the membranes is affected by the fouling or scaling. An increase in the hydrophobicity of the surface results in the increase of contact angles. Thus, fouling on membranes increases the hydrophobicity and consequently the contact angles of the membranes [22,25,26]. Considering the fact that most of the foulants are negatively charged, the fouling is a problem in the case of anion exchange membranes compared to the cation exchange membranes.

As seen, among the anion exchange membranes the highest values of contact angle on both diluate and concentrate sides were achieved when the conventional ED was utilized. The most significant differences can be seen between the contact angles on diluate side of anion exchange membranes in regime *I* compared to those of regime II and regime III, which indicates the prominent accumulation of the foulants in regime *I* onto diluate side of anion exchange membranes. The results indicate that the PER might result in a reduction of the fouling on the surface of anion

Overall, the obtained results show that the PER could decrease the fouling on the membrane surface. Consequently, the ED operation becomes more convenient and the membrane maintenance becomes more cost-effective. However, the back transfer to the diluate lowers the ED efficiency.

## 3. Materials and Methods

### 3.1. Whey

The nanofiltrated acidic whey (NFW) was obtained in curd producing and provided by the Madeta milk factory (Jindřichův Hradec, Czech Republic) which specializes in the production of milk-based desserts as well as yogurts, fermented milk products, curd and yogurt deserts. (see Table 2).

### 3.2. Reagents

The chemicals used in the experiments were of analytical grade and purchased from Sigma Aldrich (Germany). The demineralized water (қ ≤ 10 µS·cm^−1^) is produced in MemBrain Ltd., (Stráž pod Ralskem, Czech republic) by reverse osmosis.

### 3.3. Membranes

The food grade membranes were used in ED. The monopolar membranes used in ED processes for demineralization of whey were CEM-PES and AEM-PES cation and anion exchange membrane, respectively. These are heterogeneous membranes based on polyethylene as polymer and sulfonated groups as cation exchanger and quaternary ammonium groups as anion exchanger groups. Furthermore, both types of the membranes were reinforced with two polyesters (PES) fabrics. The reinforcement was performed by repressing at 150 °C and 5.06 × 10^−6^ Pa. The membranes were produced in MemBrain s.r.o., (Stráž pod Ralskem). The membranes properties including the resistivity and the permselectivity were studied and reported in previous works and presented in Table 3 [24].

The electromotive force emf method [8], was used to measure the apparent permselectivity of the membrane. To briefly explain, a two-compartment cell was used whose chambers were filled with 0.5 and 0.1 M KCl, respectively. The membrane was placed in a hole between the compartments. Two Ag/AgCl (1 M/KCl) reference electrodes were inserted into the solutions close to the membrane. After 1 h of stirring the solution with magnetic stirrers the potential between two electrodes was measured and the apparent permselectivity was calculated as a ratio of the measured potential to the theoretical potential which corresponds to a 100% permeselective membrane Equation (1):

(1) $$P\%=\;\frac{U_{measured}}{U_{theoritical}}\;\times 100$$

where (*P*) is the apparent permselectivity, (*U_measured_*) is the measured potential across the membrane and (*U_theoritica_*_l_) is the theoretical potential which is calculated for a membrane with 100% permselectivity [27,28].

For measuring the resistance of the membrane the same type of cell was used despite that both compartments were filled with 0.5 M NaCl. Two Pt wire electrodes were inserted into the solution while two Ag/AgCl (1 M/KCl) reference electrodes were placed next to the membrane on each side. The dc current of 10 mA amplitude was applied to the Pt electrodes and the resulted potential drop between the reference electrodes was measured. The same measurement was carried out without the membrane. The resistance of membrane was calculated using the ohm law, Equation (2):

(2) $$\rho_{sm}-\;\rho_{s}=\;\rho_{m}\;\left(\Omega .cm\right)$$

where (*ρ_sm_*) is the specific resistivity of the membrane and solution layers trapped between the membranes and the references electrodes, (*ρ*_s_) is the specific resistivity of the solution and (*ρ*_m_) indicates the specific resistivity of the membrane [27,28].

### 3.4. Electrodialysis

**Figure 4:**
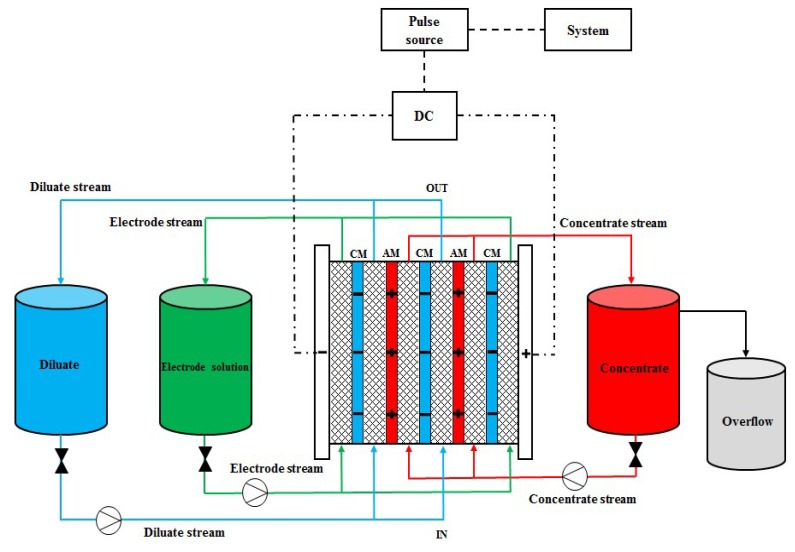
The configuration used for electrodialysis of whey.

The ED processes is shown in Figure 4. The ED was performed with modified electrodialysis unit P1 EDR-Y/50-0.8 (manufactured by MemBrain s.r.o.). The pH and the conductivity of the solutions were measured by SenTix^®^ 940 glass electrode and TetraCon 925 conductivity cell, respectively. The probes were connected to the WTW multi 3420. It must be mentioned that the conductivity cell also possesses the temperature sensor. The stack contained 50 pairs of membranes AEM-PES and CEM-PES assembled in C-A-C (cation exchange membrane–anion exchange membrane–cation exchange membrane) configuration. The active area of each membrane was 400 cm^2^. The unit was additionally equipped with a device which introduces the potential pulse and pause. The minimum length of a pulse which could be applied was 1 s. The pulse consisted of a working period and a cleaning (reverse) period. Diluate was desalinated during working period, whereas fouling was expected to be removed during cleaning period.

Fouling could be removed due to diffusion and electric migration in electric field of reverse polarity in PER. The potential in working period was 50V (1.0V/pair). Three different regimes were applied differing in the length of working and cleaning periods and the applied potential during cleaning period. Total voltage (voltage on the whole unit) and the voltage on the stack without electrode compartments were monitored. The voltage on polarizing electrodes was adjusted so that the voltage on the stack was (50 ± 1) V. In ED of whey, diluate container was filled with the 30.0 kg of nanofiltrated whey (NFW) while 7.0 kg of tap water was poured into the concentrate chamber. The flow rate and the linear velocity through the membranes of solutions are given in Table 4. The electrodes solution was 10 g·L^−1^ NaNO_3_ 7.0 kg. The ED was performed in batch mode. The ED regimes which were used for desalination of whey are shown in Table 5. To compare the effect of the different regimes on the membrane fouling two consecutive runs of each regime were performed without any cleaning step between. It must be mentioned that each regime was continued until the conductivity in diluate reached 1.0 mS·cm^−1^.

### 3.5. Fouling Analysis

The membranes samples were submitted for the SEM, immediately after the second run of each ED regime. Images were taken on an uncoated sample with a scanning electron microscope (SEM) (Quanta FEG 450, FEI, Hillsboro, OR, USA). The potential of 5 KV was applied and the working distance was 15 mm. The hydrophobicity of the membrane was studied by measuring the contact angle using (Theta QC, Attension, Espoo, Finland). For measuring the contact angle, a drop of distilled water was placed on a surface and the contact angles between the drop and the membrane surface were measured. The contact angles ranged from 0° to 180°.

### 3.6. Calculations

The Degree of demineralization in ED was obtained as Equation (3) [18]:

(3) $$Degree\;of\;demineralization\;\%=\left(1-\frac{\kappa_{\;final\;of\;diluate\;}\left(S\;m^{-1}\right)}{\kappa_{initial\;of\;diluate}\left(S\;m^{-1}\right)}\right)\times 100$$

where *(қ_initial_)* and *(қ_final_)* are the initial and final conductivity of the diluate.

Ash content %ODB (on dry basis) was calculated as Equation (4) where the ash content and the total solids are unit less parameters:

(4) $$\text{Ash content \%ODB}=\;\frac{Ash\;content\;\left(\%\right)}{Total\;solids\;\left(\%\right)}\times 100$$

The electrodialysis capacity is defined as Equation (5):

(5) $$\;C_{F}=\;\frac{m_{F}}{N.A.t\;}\;$$

where (m*_F_*) is the mass of the feed, (*A)* is the active surface of the membranes; (*N)* is the number of membrane pairs, and (*t)* is the total time of electrodialysis process.

Average ash transfer rate was determined using Equation (6):

(6) $$J\left(kgm^{-2}h^{-1}\right)=\frac{\left(m_{F}\times \;W_{F}\right)-\left(m_{D,\;final}\times \;W_{D,final}\right)}{N\times S\times t}$$

where (*m_F_)* and (*m_D,final_)* are initial and final mass of diluate, (*w_F_)* and (*w_D,final_)* are initial and final ash concentration (*g/kg*), (*N)* number of membrane pairs, (*S)* effective membrane area (*m^2^*) and *t* time (*h*).

Energy consumption was calculated Equation (7):

(7) $$E=\frac{\int_{t0}^{t1}U_{avg}Idt}{m_{F}}\sim \;\frac{\sum_{t0}^{t1}U_{avg}I∆t}{m_{F}}\;(\mathrm{Wh}/{\mathrm{kg}}_{F})$$

where (*U_avg_)* is average voltage on the stack (*V*), $\sum_{t0}^{t1}I∆t$ amount of transported charge (*Ah*) and (*m_F_)* initial weight of diluate (*kg*).

## 4. Conclusions

Comparing the conventional ED and PER in two consecutive batch experiments without cleaning in place (CIP) between them, the electrodialysis parameters are almost the same in the first and second runs of conventional ED while in PER the parameters of the second run are evidently worse than the parameters of the first run. Since CIP was not applied, deterioration of the ED parameters such as the electrodialysis capacity and the energy consumption in PER might be attributed to the fouling on the surface of the membranes and/or to the back transfer of mass during the reversal period. Considering that the SEM analysis and the contact angle values indicate that fouling on cation exchange membranes and on concentrate side of anion exchange membranes were comparable under all regimes and fouling on diluate side of anion exchange membranes was even reduced under PED regimes, it can be concluded that the back transfer to the diluate compartment when the reverse pulse was applied is dominant. However, due to the PER the fouling (scaling) was reduced in PER regimes without significant prolongation of the ED process. As shown [15] in PEF, the efficiency of the ED was improved and the fouling/scaling was decreased. However, to achieve this the time of the ED was prolonged to around 6 × that of conventional ED. In the present work, even though the parameters of the ED efficiency were slightly decreased in PER compared to the conventional ED, the duration of the ED process was only slightly increased. The decrease in ED efficiency in PER was mainly because of the back transport of minerals in reverse pulse which occurs due to the applying a high magnitude of voltage (−50 V). The back transport of minerals in a solution containing multivalent ions was also pointed out by Tufa *et al.* [29]. Despite this, in long term application the effect of the PER can be highlighted further due to a reduction of fouling and scaling on the membranes. Therefore, research must be continued to find an optimum regime in which the back transfer does not play an important role and the fouling also decreases when the optimum pulse/pause is used.

## Figures and Tables

**Table 1:** Parameters of acid whey electrodialysis with different regimes of applied potential (two consecutive runs without CIP).

Test	T (min)	κ_F_ (mS·cm^−1^)	κ_D, final_ (mS·cm^−1^)	DD (%)	J (g·m^-2^·h^−1^)	C_F_ (kg·h^−1^)	E (Wh/kg_F_)
Conventional ED 1	195	8.30	0.89	89.3	50	4.0	8.8
Conventional ED 2	195	8.31	0.88	89.4	50	4.0	8.9
180–3 50/−50	240	8.26	0.89	89.2	46	3.8	9.1
180–3 50/–50	285	8.16	0.75	90.8	39	3.2	9.6
30–5 50/–50	255	8.21	0.97	88.2	46	3.5	12.0
30–5 50/–50	315	8.23	0.97	88.3	36	2.9	12.9

**Table 2:** Feed (nanofiltrated acid whey) composition and physicochemical characteristics.

Composition	Unit	Feed Stream
Conductivity	mS·cm^−1^	8.22
pH	No unit	4.40
Total solids	%	18.6
Ash	%	1.37
Ash	%ODB	7.4
Acidity	°SH	60.0
Density	g/cm^3^	1.0794
Lactose	g·kg^−1^	143.1
Total proteins	g·kg^−1^	14.9
True proteins	g·kg^−1^	5.3
NPN	g·kg^−1^	1.5
α-LA	g·L^−1^	1.46
β-LG A	g·L^−1^	2.40
β-LG B	g·L^−1^	0.62
CMP	g·L^−1^	2.63
Lactates	mg·kg^−1^	15706.87
Citrates	mg·kg^−1^	7014
Na^+^	mg·kg^−1^	362.79
K^+^	mg·kg^-1^	1404.65
Mg^2+^	mg·kg^−1^	320.93
Ca^2+^	mg·kg^−1^	3106.97
S	mg·kg^−1^	221.39
P total	mg·kg^−1^	1925.58
Cl^-^	mg·kg^−1^	812.09
NO_3_	mg·kg^−1^	4.65

**Table 3:** Properties of the membranes used in ED.

Membrane	d^1^ Dry (mm)	d Swallowed (mm)	$\rho^{2}\;(\Omega \cdot cm)$	P^3^ (%)
AEM-PES	0.45	0.75	120	>90
CEM-PES	0.45	0.70	120	>95

^1^d: the thickness of the membrane; ^2^*ρ*: specific resistivity; ^3^P: the apparent permselectivity.

**Table 4:** Process conditions.

Parameter	Unit	Diluate	Concentrate	Electrolyte
Utilized solution	-	Acidic whey	Tap water	Sodium nitrate
Concentration	%	20.0	-	1.0
Initial mass	kg	30.0	7.0	7.0
Solution flow rates	L/h	700	700	500
Thickness of spacers	mm	0.8	0.8	1.0
pH	-	4.4	5.5	3.0
Ending status	mS·cm^−1^	1.1	15.0	-
Temperature	°C	15 ± 2	15 ± 2	15 ± 2

**Table 5:** The ED regimes which were applied for the desalination of whey.

Electrodialysis	Working Voltage	Reverse Voltage	Working Period	Reverse Period
Regime I (conventional ED)	50	Not used	Not used	Not used
Regime II	50	−50	180 s	3 s
Regime III	50	−50	30 s	5 s

## Supplementary Materials

Supplementary materials can be found at https://www.mdpi.com/1422-0067/20/8/1918/s1.

## Abbreviations

*U*/V	Cell voltage
*m*/kg	Mass
*A*/m^2^	Membrane geometric surface area
*N*	Number of membrane pairs
*I/A*	Current
*P*	Apparent permselectivity
*m_F_*/ kg	Mass of the feed in electrodialysis
*N*	Number of moles
*t/s*	Time
*F*/96500 C·mol^−1^	Faraday constant
*к*/mS·m^−1^	Specific conductivity
*j_i_*/mole. s^−1^·m^−2^	Mass flux of i through the membrane
*C_F_*/kg·m^−2^·h^−1^	Electrodialysis capacity
g/cm^3^	Density
*E* Wh·kg_F_^−1^	Electrical energy used in electrodialysis per mass of the feed
α-LA	α-lactalbumin (whey protein)
β-LG A	β-lactoglobulin A (whey protein)
β-LG B	β-lactoglobulin B (whey protein)
AEM	Anion exchange membrane
CEM	Cation exchange membrane
CMP	Casein macropeptide
CIP	Cleaning in place
°C	Temperature
DD	Degree of demineralization
ED	Electrodialysis
F	Feed (raw material)
NFW	Nanofiltered acidic whey
ODB	On dry basis in percent (Ash)
P	Product
PED	Pulsed electrodialysis
PER	Pulsed electrodialysis reversal
°SH	Acidity, Soxlet Henkel degrees (0.25N NaOH)
