# Supplementary material for: An Investigation on the Application of Pulsed Electrodialysis Reversal in Whey Desalination
Source: Int J Mol Sci. 2019 Apr 18;20(8):1918. doi: 10.3390/ijms20081918 (PMC6515537; doi:10.3390/ijms20081918)
Supplement: Supplementary file 1 [file ijms-20-01918-s001.pdf]

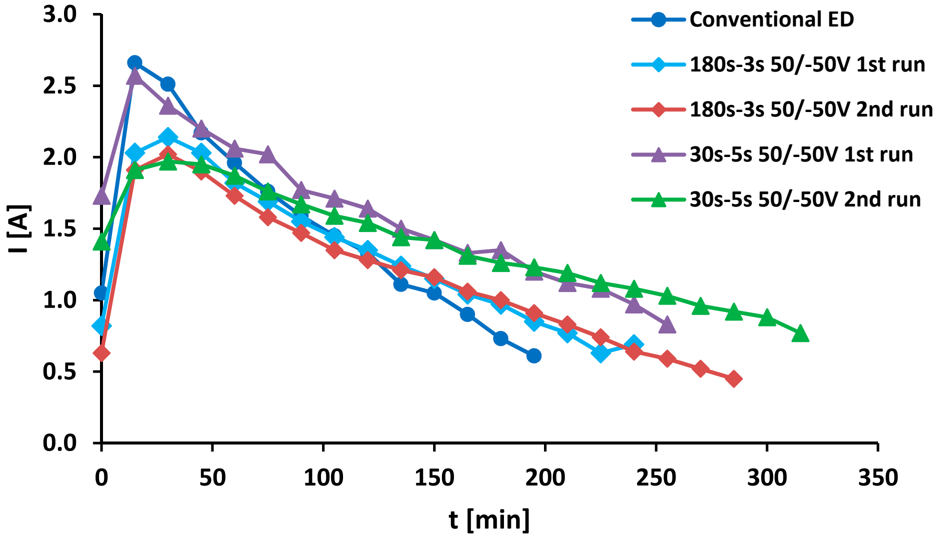

Figure 1. Current during ED desalination

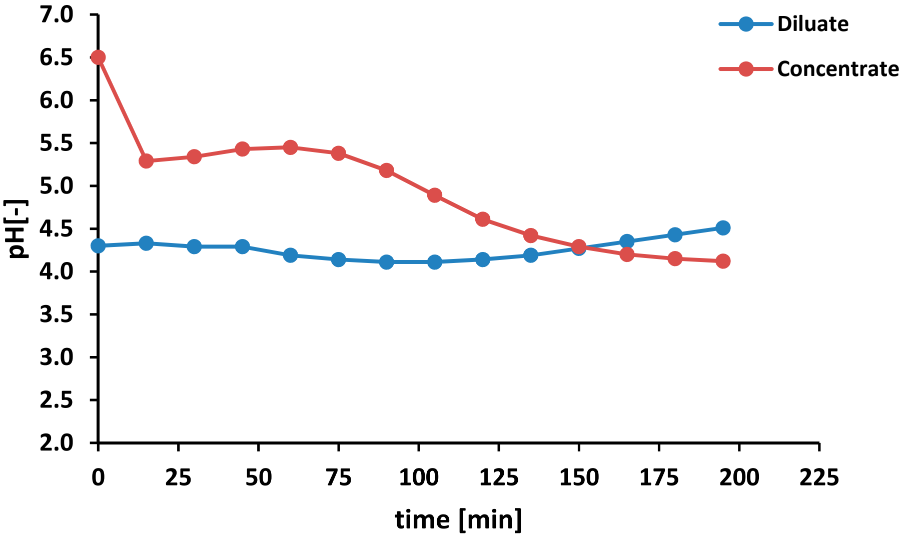

Figure 2. Diluate and concentrate pH of acidic whey during conventional ED desalination
